# The mother during pregnancy and the puerperium: Detailed data abstracted from the clinical obstetric records of ALSPAC pregnancies

**DOI:** 10.12688/wellcomeopenres.16603.2

**Published:** 2024-03-26

**Authors:** Karen Birmingham, Steven Gregory, Yasmin Iles-Caven, Abigail Fraser, Deborah A. Lawlor, Andrew Boyd, Kate Northstone, Jean Golding

**Affiliations:** 1 Bristol Medical School (PHS), University of Bristol, Bristol, BS8 2BN, UK

**Keywords:** ALSPAC, Pregnancy, Obstetric care, Labour, Delivery, Postpartum

## Abstract

**Background:**

When the Avon Longitudinal Study of Parents and Children (ALSPAC) was planned, it was assumed that the clinical obstetric data would be easily accessible from the newly developed National Health Service computerised ‘STORK’ system. Pilot studies, however, showed that, although fairly accurate in regard to aspects of labour and delivery, it was, at the time (1990-2), inadequate for identifying the full antenatal and postnatal details of clinical complications and treatments of the women in the Study.

**Methods:**

A scheme was therefore developed to train research staff to find and abstract relevant details from clinical records onto proformas designed for the purpose. Extracting such data proved very time consuming (up to six hours for complicated pregnancies) and consequently expensive. Funding for the enterprise was obtained piecemeal using specific focussed grants to extract data for subsamples of the Study, including a random sample to serve as controls.

**Results:**

To date, detailed records have been completed for 8369 pregnancies, and a further 5336 (13,705 in total) have complete details on specific prenatal areas, including serial measures of maternal blood pressure, proteinuria and weight. In this Data Note we describe the information abstracted from the obstetric medical records concerning the mother during pregnancy, labour, delivery and the first two weeks of the puerperium. Information abstracted relating to the fetus (including fetal monitoring, presentation, method of delivery) and neonate (signs of asphyxia, resuscitation, treatment and well-being) have been described in a further Data Note.

**Conclusions:**

These data add depth to ALSPAC concerning ways in which the signs and symptoms, procedures and treatments of the mother prenatally, intrapartum and postnatally, may impact on the long-term health and development of both mother and child. They augment the data collected from the mothers’ questionnaires (described elsewhere) and the ‘STORK’ digital hospital data.

## Abbreviations

**ALEC**   ALSPAC Ethics and Law Committee

**ALSPAC**   Avon Longitudinal Study of Parents and Children

**BMH**   Bristol Maternity Hospital

**CS**   Caesarean section

**CVS**   Chorionic villus sampling

**ECV**   External cephalic version

**EDD**   Estimated date of delivery

**EPDS**   Edinburgh Postnatal Depression Scale

**GP**   General practitioner

**Hb**   Haemoglobin

**IVF**   In vitro fertilisation

**LMP**   Last menstrual period (date of)

**LREC**   Local Research Ethics Committee

**MRC**   Medical Research Council

**NHMRC**   National Health and Medical Research Council of Australia

**NHS**   National Health Service

**NIH**   National Institutes of Health, USA

**PI**   Principal investigator

**PLIKS**   Psychosis Like Symptoms

**R & D**   Research and development

**STD**   Sexually transmitted disease

**TENS**   Transcutaneous electrical nerve stimulation

**WGH**   Weston General Hospital

**WHO**   World Health Organisation

## Introduction

### The rationale

The Avon Longitudinal Study of Pregnancy and Childhood (ALSPAC - later renamed Avon Longitudinal Study of Parents and Children) was specifically designed to identify, *inter alia,* the possible adverse or beneficial effects of environmental features on the development and health of the child. Particular attention was paid to the pregnancy and the first year of life; the environment was defined as anything (other than genes) that might have an effect, as the developing fetus was especially sensitive to change during this period. Consequently, as pregnancy was known to be important for development of organs such as the brain, it was decided to obtain as much information as possible for this period. It was clear that the pregnant woman herself was best able to report on various environmental exposures to herself when at home, but it was not clear that she would be able to report accurately on the clinical progress of her pregnancy, including the investigations, diagnoses, medications and other treatments she may have undergone as part of antenatal and inpatient care.

### Designing the data collection tool

There were a number of influences on the design of the data that have been collected. These were partly based on reviews of the literature to identify factors thought to influence the development of the child, and information collected in other studies of birth cohorts (e.g. the 1958 and 1970 National UK birth cohort studies), but the major influences (particularly in regard to recording data relevant to the hypertensive disorders of pregnancy) were those developed by Jean Golding and colleagues for (a) the International Study of the Hypertensive Disorders of Pregnancy funded by WHO (World Health Organisation), which took place in a number of developing countries including Thailand, Vietnam and China ( Golding *et al.*, 1988); and (b) The Jamaica Low Dose Aspirin Study (Golding *et al.*, 1998).

The latter studies showed the importance of collecting the actual measurements (e.g. of blood pressure, proteinuria, haemoglobin) rather than relying on clinical diagnoses which were likely to vary with the clinician/hospital/country. This background dictated the importance of abstracting as many measurements as possible, as well as details of investigations and treatments. The data abstraction form ( Birmingham *et al.*, 2021a), with the instructions to the Data Abstractors ( Birmingham *et al.*, 2021b) and checkers ( Birmingham *et al.*, 2021c) have been made available (see *Extended data*). 

### Structure of the obstetric services in Avon 1990-2

The Study area (defined as that part of the county of Avon within the South West Regional Health Authority, hereinafter referred to as Avon ( Boyd *et al.*, 2019)) was served by consultant obstetric services based in the two major obstetric teaching hospitals within Bristol: Southmead Hospital and the Bristol Maternity Hospital (BMH) (subsequently known as St Michael’s Hospital). In addition, deliveries of low-risk pregnancies were undertaken at Weston General Hospital (WGH) situated in Weston-super-Mare. There were dedicated neonatal intensive care and special care baby units at BMH and Southmead but not at WGH. All pregnancy and delivery care was free as part of the National Health Service (NHS), and private practitioners were used rarely by the Avon maternity population. 

Antenatal care was undertaken by general practitioners (GPs) and community midwives. For most pregnancies, one of the consultant obstetricians would also have been involved and, provided he/she was happy that the woman was not of very high risk, she would have had “shared care”. Very few women intended to have a home delivery.

Thus, for women enrolled in ALSPAC, the system for antenatal care in those at low risk involved shared care between hospital-based consultant obstetricians and midwives based in the community and working with the woman’s GP. Women at high risk of complications would be more likely to have been seen throughout their pregnancy by the clinical obstetric services, mainly within the relevant hospital, although some obstetricians visited community clinics, particularly those based in areas of deprivation. 

The contemporary protocols concerning care used by the medical staff at each of the three hospitals have been added to the ALSPAC archive as part of the University of Bristol Special Collections [Box 784]. These protocols should be used with caution as there is evidence that these guidelines were not always adhered to.

The hand-written clinical measurements and observations made in the community and those made in hospital were all paper records and filed by each hospital in a single folder, with the exception of fertility and psychiatric records, which were kept separately. Important information could be found in many different places within the records, having been documented variously by medical and nursing/midwifery staff. This required meticulous systematic scrutiny in order to abstract accurate data. Details of all pregnancy-related hospital admissions were also included in the record. For complex pregnancies the folder could be as thick as 6–8 inches, comprising A4-sized paper, frequently handwritten on both sides, as well as laboratory results on flimsy print outs.

The STORK digital record of pregnancy and delivery had been initiated in the two major hospitals shortly before the start of the enrolment of the pregnant women in ALSPAC. Comparison of the data collected with the information desired showed that the information in the computerised record was reasonably accurate for many features of labour and delivery, but it lacked the fine detail, particularly in regard to antenatal and postnatal information - including the repeat measures such as of weight and blood pressure. The ALSPAC team reluctantly decided therefore to collect all relevant data by hand from the medical records.

## Methods and materials

ALSPAC was designed to assess the ways in which the environment interacts with the genotype to influence health and development ( Boyd *et al.*, 2013; Fraser *et al.*, 2013). Pregnant women resident in the Study area with an expected date of delivery between 1 ^st^ April 1991 and 31 ^st^ December 1992, were invited to take part. About 80% of the eligible population did so. The initial ALSPAC sample consisted of 14,541 pregnancies; of these initial pregnancies, there was a total of 14,676 fetuses, resulting in 14,062 live births and 13,988 children who were alive at one year of age. Information on the cohort parents and their offspring was collected using a variety of methodologies including self-completion questionnaires sent to Study mothers, fathers, teachers and the Study child, direct examination under standardized conditions, and linkage to other medical information from NHS records and educational data from the school systems. Please note that the Study website contains details of all the data that is available through a fully searchable data dictionary and variable search tool ( http://www.bristol.ac.uk/alspac/researchers/our-data/).

Ethical approval for the study was obtained from the ALSPAC Ethics and Law Committee (ALEC; IRB00003312) and the NHS Local Research Ethics Committees (LREC) ( Birmingham, 2018). Detailed information on the ways in which confidentiality of the cohort is maintained may be found on the Study website: http://www.bristol.ac.uk/media-library/sites/alspac/documents/pearl/CO90S_linkage_detailed.pdf and details of all NHS Research Ethics approvals: http://www.bristol.ac.uk/media-library/sites/alspac/documents/governance/Research%20Ethics%20Committee%20approval%20references.pdf.

### The subsample strategy

Unfortunately, for the first 10 years of the Study there was no designated core funding available, and ALSPAC survived by including a ‘fee’ in each grant application to contribute towards the funding of the central running of the Study. The initial funding obtained for ALSPAC was used for costs that could not be postponed: collection of contemporary information from mothers and their partners, as well as for processing and storage of biological samples. The obstetric records could be accessed and abstracted as and when funding became available. Although unavoidable, there was a major disadvantage with this strategy in that the relevant hospitals became short of storage space for records, and they were, by 2006, held on 19 different sites. Consequently, searching for any particular record took an average of two hours.

In parallel, some obstetric records were also stored in non-paper formats (optical discs or microfiche). Electronic tracing of hospital records was inefficient, with the systems frequently inaccurate and not kept up to date. Extensive knowledge of both the computer systems and physical environment was necessary to locate the records. Double or triple registration with equivalent sets of notes was not uncommon after a merger of two of the hospitals’ records departments.

Data abstraction is expensive primarily because it is extremely time consuming. ALSPAC was unable to obtain funding to extract the full set of detailed obstetric records relevant to all pregnancies in the Study. The fall-back position was to use parts of grants for specific projects and consequently the abstraction of many of the obstetric and neonatal records was funded using three types of funding sources: (a) specific project grants for the purpose; (b) as a small part of the ‘core funding’ from the Medical Research Council (MRC) and the Wellcome Trust after the year 2000, and (c) using portions of the ‘ALSPAC fee’ of the project grants obtained for ALSPAC in the early years of the Study. Below are listed the eight grants with funding for specific abstraction from the obstetric records.

(1) Twins in a natural experiment to study causes of language delay; Jean Golding (PI), Michael Rutter, Karen Thorpe; Funder: Mental Health Foundation.
(2) Twins in a natural experiment to study causes of language delay; Jean Golding (PI), Karen Thorpe, Sue Roulstone; Funder: South West Regional Health Authority R & D.
(3) The association between different types of antenatal care and the mother’s anxiety and depression levels; Jean Golding (PI), David Jewell, Lindsay Smith, Ian MacGillivray, Helen Francomb; Funder: South & West Regional Health Authority.
(4) Obstetric and medical consequences of teenage pregnancy; David Jewell (PI); Jean Golding; Funder: NHS Executive South & West R & D.
(5) Evidence of the long-term consequences of caesarean section is required to allow informed maternal choice; Gordon Stirrat (PI), Jean Golding; Funder: Bupa Foundation.
(6) Fetal loss in a multiple pregnancy: a possible cause of cerebral palsy; Peter Pharoah (PI), Department of Public Health, University of Liverpool, Bristol co-applicants: Helen Porter, Jeremy Berry, Jean Golding, Alan Emond; Funder: Children Nationwide/National Lottery
(7) Factor V Leiden and adverse pregnancy outcome: An ALSPAC nested case-control study; Rodney Scott and Tracey Dudding, University of Newcastle, New South Wales, Australia; Funder: NHMRC via University of Newcastle, Australia.
(8) Investigating genetic and epidemiological risk factors for sub-clinical psychosis-like symptoms (PLIKS) in a birth cohort study; Stan Zammit (PI); Funder: Department of Health National Clinical Scientist Award.

Publications as the result of these grants are available ( Dudding *et al.*, 2008; Patel *et al.*, 2005a; Patel *et al.*, 2005b; Rutter *et al.*, 2003; Thorpe *et al.*, 2003; Zammit *et al.*, 2009), together with other publications specifically focussed on different subgroups.

### Definition of the subsamples

As a result of the funded grants and the funding restrictions, to date only 8369 pregnancies have undergone selection for the detailed data abstraction using the proforma shown in the data abstraction form (see *Extended data* ( Birmingham *et al.*, 2021a)). The different selection criteria are described below.

(i) Twins and closely spaced singletons: This study compared the development of twin pairs with singletons with a closely spaced sibling. Abstraction of the obstetric and neonatal records was to test whether delay in twin development of speech was related to obstetric problems in pregnancy or whether it concerned the difficulties mothers had in relating to two similarly aged siblings; 94 twin pregnancies were compared with 97 pregnancies where there was a sibling born within 30 months of the Study child ( Rutter *et al.*, 2003).
(ii) All multiple pregnancies: After completion of the selection of the twins for the Rutter study, it was decided for completion to extract details of all remaining multiple pregnancies, regardless of the outcome of pregnancy (n = 188 in total).
(iii) All fetal and neonatal losses: These data will be described in the neonatal Data Note.
(iv) Cerebral palsy and missing twins: The children with diagnosed cerebral palsy were identified through the Community Child Health system. Only those with a Study placenta available were selected. This was part of the study headed by Peter Pharaoh (see above) to determine whether there was any evidence of a missing twin among children with cerebral palsy.
  In addition, we identified from the maternal questionnaires administered at 18 weeks gestation and two months post-delivery all those instances where the mother had indicated that, during pregnancy, she had been informed that she had, or might have had, a multiple pregnancy, but in fact she delivered a singleton. [The placentas of all in this group (n=67) were examined for signs of a missing twin as were their controls (n=124) and children with cerebral palsy who had placentas available (n=18)]. The results were largely negative and never published.
(v) Delivered preterm: In order to determine an accurate preterm delivery rate within ALSPAC, all deliveries with gestation <37 weeks identified using any of: mother’s stated estimated date of delivery (EDD), EDD based on date of last menstrual period (LMP), gestation on STORK, and obstetrician’s estimates of gestation were considered. If there was consistency between two or more records, the records were not selected for review and the delivery was judged to be preterm. If some estimates were <37 completed weeks and others were not, the records were obtained and the data abstracted, and a decision made by a consultant obstetrician using the clinical information in the notes, including the results from early ultrasound scans. This resulted in identification of two groups of pregnancies – one of definite preterm delivery (those of <36 weeks agreed by all sources; n = 480), and the other of possible preterm delivery upon which the decision had been made (<37 completed weeks n = 1022). These data were used to identify the ALSPAC preterm delivery rate as 5.5% ( Little *et al.*, 2004).
(vi) Teenage mothers: ALSPAC pregnancies to women aged <20 years at the time of delivery (n = 645).
(vii) Mothers with depression: This sample was selected to include women who were either depressed (defined as having a score of greater than 12 on the Edinburgh Postnatal Depression Scale (EPDS; Cox *et al.*, 1987) completed by the mother during pregnancy and/or at eight weeks post-delivery (n = 1426).
(viii) Caesarean section (CS): Pregnancies resulting in caesarean sections were identified from the mothers’ questionnaires (at eight weeks, n = 1198) augmented with cases from the computer system STORK, which covered the two major hospitals BMH and Southmead (total n = 1473).
(ix) Instrumental vaginal deliveries: Pregnancies resulting in instrumental vaginal deliveries (i.e. using forceps or vacuum extraction) were identified from STORK (n = 1521). Consequently, deliveries outside the two major hospitals but who had instrumental deliveries will not have been selected unless picked up in other selections (e.g. in (xii) below).
(x) Attended Children in Focus Research Clinic: Selection of children included in Children in Focus ( ~ 10% sub-sample of children born in the last six months of 1992), used a quasi-random method (if the day of the mother’s birth was an odd or even number determined whether or not she would have been eligible). The sample chosen comprised those who were eligible and attended at least one of the 10 clinics for hands-on measurements (n = 1377).
(xi) Had symptoms of psychosis like symptoms (PLIKS): At age 12 the children were interviewed in regard to a number of psychosis like symptoms. If they were positive on any of the signs, the delivery records were abstracted (n = 870; see Zammit *et al.*, 2009 for details).
(xii) Deliveries outside of Avon hospitals: This group includes home deliveries, babies born before arrival at the hospital or delivered in hospitals outside the Avon area. These records were abstracted since it was likely that they would be difficult to find in the future (n=352).
(xiii) Random sample: The random sample was selected from the complete set of ALSPAC births for the depression study by the statistician Jon Heron. This selection was expanded as the other sections were completed. The selection includes (but is not confined to) pregnancies that appear within other sections (n=2760).

It is important to note that, in general, the data abstractors were not aware as to which group the pregnancy was in. This of course would have been obvious to them once they had accessed the medical records for selections on the type or place of delivery, teenage pregnancies, twins or preterm deliveries, but selections based on maternal depression or specific child outcomes were not revealed to them and they were blind as to whether the records concerned a specified group or the random control group.

### Abstraction and checking of information

As already noted, the data abstractors used a paper proforma which was identical for all subgroups (with the exception of the twin sample) on which to detail the information from the medical records ( *Extended data* ( Birmingham *et al.*, 2021a)), rather than keying straight onto a digital form. This was because, for the most part, the medical records had little structure, particularly for women with complex histories including several hospital admissions during the pregnancy. Key pieces of information could be found in various entries and could be contradictory. Thus, the team who carried out the data abstraction had to be skilled in the recognition of source and validation of various items of information. They mainly had a background in midwifery and/or nursing. The relevant instructions to the data abstractors (including how to resolve contradictory information); definitions of the items to be recorded are shown in *Extended data* ( Birmingham *et al.*, 2021b). Each completed form was meticulously checked by another data abstractor without reference to the original records; instructions for such checks are shown in *Extended data* ( Birmingham *et al.*, 2021c). If queries or inconsistencies were unresolved, the records would be referred to again. These checks were made before the record folders were returned to the hospital record stores to prevent having to search for them a second time. 

Once the forms had been completed and checked, the information was double keyed by an external bureau. There were two types of data – the data for which boxes had been ticked or numbers filled in (such as dates and the results of tests), and other details that were keyed in-house as text. The text is available for coding by those with particular interests, on application to the ALSPAC Executive. The more specific information is available and is summarised in *Summary of the data available*.

### The variable numbering system

 The variable numbers for most of this data set all start with the letters ‘DEL_P’ followed by a number. For simplicity this will be known as the P number throughout this paper. In addition, the question number is quoted – i.e. the actual question asked on the data abstraction form ( *Extended data* ( Birmingham *et al.*, 2021a)), as well as to the instructions for the data abstractors, reproduced as *Extended data* ( Birmingham *et al.*, 2021b). The variable nomenclature differs, however, for data abstracted for the whole dataset (see *The extended dataset abstraction*), for the ultrasound scans (see *Ultrasound scans*) and the antenatal admissions (see *Antenatal hospital extractions*) although the form and instructions remain valid.

### Suggested statistical analysis using the subgroup design

 For analysis of the 8369 pregnancies, it is important to note the potential biases in ascertainment of some of the subgroups (see *Definition of the subsamples*). There are a number of alternative ways in which these could be addressed in statistical analyses, as suggested briefly below.

(a) If there is sufficient power, we recommend using the random sample only.

(b) An alternative is to include, with the truly random sample the randomly ascertained group of pregnancies with livebirths followed up as Children in Focus.

or

(c) Combine all the groups and use conditional or unconditional analytic strategies taking account of the different groups (see Pearce (2016) for discussion of pros and cons).

### Omissions from this Data Note

 In this paper we have omitted information from the obstetric data abstraction that relates to the fetus or neonate. That information is described in a separate Data Note ( Birmingham *et al.*, 2021d) which concentrates on the details relevant to the fetus, the birth and the neonatal period. Thus, it describes separate records for each member of a multiple pregnancy, and includes details of fetal distress and gestation at delivery. 

### The extended dataset abstraction

Two grants awarded in 2006 (US National Institutes of Health [NIH]) and 2009 (Wellcome Trust) to DA Lawlor and colleagues provided funds to complete abstraction of certain data from the clinical obstetric records from all remaining eligible women. The selected data comprised ABO and Rhesus blood groups, and repeated measurements of weight, blood pressure, glycosuria, haemoglobin and proteinuria, but did not include many other antenatal, intrapartum and postnatal measurements, treatments or procedures. With this effort, data became available on a total of 13,706 women (13,899 offspring) with derived variables on hypertensive disorders of pregnancy, gestational diabetes, anaemia, and maternal weight gain. For analyses of these data there is no need to use the strategies described under *Suggested statistical analysis using the subgroup design* since these data are available on the whole sample ( http://www.bristol.ac.uk/alspac/external/documents/ALSPAC_Data_Dictionary.zip ^1^; see also Lawlor *et al.*, 2010; Macdonald-Wallis *et al.*, 2012; Macdonald-Wallis *et al.*, 2014; Macdonald-Wallis *et al.*, 2015).

## Summary of the data available

In this section we describe most of the data available on the ALSPAC Study pregnancies. For further details of the data see the file in the ALSPAC Data Dictionary:

http://www.bristol.ac.uk/alspac/external/documents/ALSPAC_Data_Dictionary.zip ^2^.

### Serial measures during pregnancy

***Ultrasound scans.*** At the time when the Study mothers were enrolled there was considerable discussion as to whether ultrasound scans were completely safe for the developing fetus – particularly for the child’s subsequent development ( Reece *et al.*, 1990). For this reason, details of all ultrasound scans were collected. Data collected for each scan included: (a) gestation at which the scan was performed (calculated using the date of scan and the best clinical estimate of the EDD); (b) the reason for the scan; (c) the type of scan; and (d) the results of the scan ( Table 1).

**Table 1.** The variables used and numbers of responses for each ultrasound scan.

Scan No.	Variables DEL_	No. with gestation	No. with type	No. with Reason	No. with Result	No. “Abnormal”
1	S010-3	7945	5214	7236	8103	3684
2	S020-3	5491	3965	5186	5362	2562
3	S030-3	3013	2063	2828	3014	1349
4	S040-3	1712	1275	1613	1704	789
5	S050-3	1034	798	984	1025	491

[Admissions 6–33 are similarly available.]

Among the 8369 women for whom the medical records data have been fully abstracted, 7945 (95%) had documentation for up to 33 scans occurring before the baby was born. The scans included those carried out in the community (usually in GP surgeries), in outpatient radiography clinics, and as part of inpatient care. All scans undertaken between conception and delivery were included. Any postpartum scans were excluded. 

 Type of scan was coded as: (A) Clinic scan; (B) Dating scan; (C) Departmental scan; (D) Doppler scan; (E) Follow-up scan; (F) Mini-scan; (G) Private scan; (H) Real time scan; (I) Routine scan; (J) ‘Survey scan’; (K) Trans-vaginal scan. Reasons for each scan were coded by the data abstractors with a coding schema developed by ALSPAC. The 27 codes used comprised: (1) Maternal abnormality (e.g. fibroids); (2) Amniocentesis; (3) Biophysical profile; (4) Bleeding; (5) Choroid plexus seen on previous scan; (6) Chorionic villus sampling (CVS); (7) Dates; (8) Fetal anomaly; (9) Fetal growth; (10) Fetal movements; (11) Multiple pregnancy; (12) Pelvimetry; (13) Placental location; (14) Presentation of baby; (21) More than one reason; (22) Volume of liquor; (23) Pre-eclampsia symptoms queried; (24) Fetal well-being; (25) Viability; (26) As part of fertility regime; (27) Suspected fetal abnormality. It should be emphasised that these categories of reasons were defined by what was written in the notes – the data abstractors were instructed not to assume, or guess.

 The result of the scan was characterised as normal or abnormal. ‘Normal’ was coded if nothing out of the ordinary was noted, and ‘Abnormal’ if there was something of importance picked up on the scan including: breech or transverse presentation; date change by more than one week; low lying placenta; or presence of two or more fetuses (once identified, any subsequent scans that merely confirmed twins were coded as normal). The descriptions of the abnormalities were described as text and have not been coded yet.

***Antenatal hospital admissions.*** This section did not include admissions of mothers who were in labour on admission, only admissions during pregnancy before the onset of labour. In all 5715 women were admitted to hospital at some point before the start of labour (DEL_H050). The total no. of admissions per woman varied from 0 to 18 (DEL_H002), and the no. of days in total that the mothers stayed as an inpatient ranged from 1 to 80 (DEL_H001).

For each admission, coded information was collected on three items: the gestation at admission (in days), the number of days stayed and the hospital involved ( Table 2). The data abstractors were instructed to record as text the reasons for each admission together with details of any treatment (including medications) given. The text has been keyed (but not yet coded) and is available on request. Results of standard measurements taken in hospital (blood pressures, weight, etc) have been added to the serial measurements (see *Serial antenatal measurements made*). The instructions were to include only one measurement per day and, if there were more, to select the one with the highest diastolic blood pressure.

**Table 2.** Table showing the variable numbers for the first nine admissions, the range of gestations at admission, the range in the number of days stayed, and the number of pregnancies involved.

Admission no.	Variables DEL_	No. pregnancies	Gestation at admission	Days stayed
1	H051-3	5651	1-306	1-54
2	H054-6	2460	34-305	1-70
3	H057-9	1039	65-303	1-49
4	H060-2	462	74-302	1-58
5	H063-5	204	103-302	1-18
6	H066-8	108	125-299	1-17
7	H069-71	44	186-300	1-17
8	H072-4	24	217-301	1-8
9	H075-7	13	219-301	1-7

[The admissions 10–18 continue in a similar format.]

***Serial antenatal measurements made.*** Measurements made at each antenatal visit and inpatient admission were recorded ( Table 3); those made during labour and the postpartum period were noted elsewhere (see *Labour and delivery* and *The mother in the puerperium*). The measures recorded for each antenatal event include: (a) gestation in days; (b) place at which measurements were made, distinguishing between (i) community care, (ii) inpatient care, (iii) home visit, and (iv) consultant clinic; (c) maternal weight; (d) systolic and diastolic blood pressure; (e) proteinuria; (f) sites of oedema (if any); (g) haemoglobin level; and (h) level of glycosuria ( Table 3). The actual data for each measurement is described in more detail in http://www.bristol.ac.uk/alspac/external/documents/ALSPAC_Data_Dictionary.zip ^1^ and includes the extra data abstracted by Deborah Lawlor and team (see *The extended dataset abstraction*).

**Table 3.** Numbers of pregnancies at each measurement event for which the following measurements were taken: A – place taken; B – gestation; C – maternal weight; D – blood pressure; E – level of proteinuria (recorded as nil; trace; +; ++; +++ or more; blood); F – oedema (coded as none; ankles only; hands only; face only; generalised; more than one site; and not otherwise stated (n.o.s)); G – level of haemoglobin (in g/dL); and H – level of glycosuria (recorded as nil; trace to +; ++; +++ or more; 0.25%; 0.5%; 1% or more). The first five of 49 possible measurement events.

Visit No.	Var nos.	No. A	No. B	No. C	No. D	No. E	No. F	No. G	No. H
1	*1	13548	13729	8944	7788	5420	10515	10011	5418
2	*2	13463	13541	11648	11828	10381	12868	1904	10379
3	*3	13279	13352	11697	12328	10934	12907	1003	10927
4	*4	13148	13209	11726	12499	11289	12845	1176	11292
5	*5	13050	13071	11641	12536	11541	12800	2301	11531

[The variable number for each measurement varies with the number of the measurement as shown above. For example, the systolic blood pressure is given by ‘v1dab1g_systolic_bp*’ where the asterisk denotes the event number above.]

This data set has the advantage of using all the medical records that could be retrieved, and consequently can be used to identify prevalence. Lawlor’s group have derived a number of useful variables including (a) maternal weight change between 0–18 weeks gestation (DEL_1128), (b) maternal weight change 18–28 weeks gestation (DEL_1129), maximum level of glycosuria (DEL-1034), and first haemoglobin measurement (DEL_ 1047). For further information on this complete data set see: http://www.bristol.ac.uk/alspac/external/documents/ALSPAC_Data_Dictionary.zip ^1^

### Features of pregnancy

***Conception.*** Although data were collected on whether the pregnancy had resulted from fertility treatment, most of this information is coded as text, with the exception of *in vitro* fertilisation (IVF), which has been coded. Other sources of information on fertility treatment can be obtained from the maternal questionnaire D (for example variable D031 documents the methods used to help the woman conceive, and D032 identifies ovulation induction). The actual date of last menstrual period (LMP) was not recorded and is not available for confidentiality reasons. However, it was used for calculations of the various stages of gestation when events occurred. In contrast the month of LMP is available for analysis of seasonal effects. Whether or not the woman was certain of the date of her LMP was collected as an indicator of her personality as well as of a possible problem for the obstetric team ( Table 4). 

**Table 4.** Features concerning the conception.

Information	P no.	Q no.	N with Data	N with criteria
IVF conception	1000	B65	8369	63
Month of LMP	1004	B2amm	8218	8218
Certain of LMP	1005	B2b	8178	6737

[N.B. The extended abstracted dataset also includes these data.]

***Planning for obstetric care.*** The type of antenatal care planned is given in DEL_P1003. Of the 8307 pregnancies with this information, 8089 (97%) were planned to have shared care, and only 218 had a different initial plan. At the first antenatal visit a plan was also made as to where the baby would be delivered. Table 5 summarises the initial intention compared with where they actually were delivered.

**Table 5.** The intended and actual place of delivery.

Place of delivery	Intended	Actual
	DEL_P001	DEL_P002
BMH	3163	3189
Southmead	4486	4399
Weston General	457	369
Home	64	207
Other	99	169

***Medical complications of pregnancy.*** Abstracted from the medical records were a number of specific conditions. The ALSPAC codes and numbers of women involved are shown in Table 6. It should be noted that a further source of information on bleeding, infections and other problems can be found in the maternally completed questionnaires B, C and E, which detail the episodes by trimester (see Ellis *et al.*, 2022).

**Table 6.** Medical complications during pregnancy.

Medical history	P no.	Q no.	No. with Data	No. with condition
*History of bleeding during pregnancy*				
1 ^st^ trimester	1040	B6jj	8369	935
2 ^nd^ trimester	1041	B6kk	8369	415
3 ^rd^ trimester	1042	B6ll	8369	647
Abruption	1044	B6w	8369	27
Threatened abortion ^#^	1044	B6dd	8369	136
Placenta praevia ^#^	1045	B6x	8369	81
Any of above	1046	derived	8369	1752
*Infections noted in pregnancy*				
Genital herpes	1050	B6p	8369	29
Other STD	1051,2	B6q,B6cc	8369	<5
Urinary tract infection	1053	B6hh	8369	510
Vaginal infection	1054	B6mm	8369	1229
Hepatitis B	1055	B6r	8369	<5
*Diagnoses made by the clinicians involved*				
Anaemia *	1060	B6c	8369	1052
Hyperemesis *	1061	B6m	8369	86
Diabetes *	1030	B6k	8369	91
Eclampsia	1022	B6i	8369	<5
Hypertensive disorders of pregnancy *	1020	B5	8175	668
*Other complications of pregnancy*				
Oligohydramnios	1103	B6u	8369	117
Polyhydramnios	1104	B6y	8369	114
Multiple pregnancy ^a^	1107	B8a	8279	221
Threatened preterm labour ^b^	1106	B6ee	8369	592

^#^Instructions were to code whether or not accompanied by bleeding; ^a^not necessarily a multiple delivery; ^b^this was recorded even if ultimately delivered preterm; *the extended abstracted dataset also has this information <5 may equal 0.

***Results of standard laboratory tests during pregnancy.*** The ABO and Rhesus blood groups of the women are shown in Table 7, and the presence of Rhesus antibodies, together with other biochemical results in Table 8.

**Table 7.** ABO and Rhesus blood groups (including extended abstracted dataset).

Blood group	Variable	n	%
ABO	v1dab3a_abo		
- A		5839	43.0
- B		1225	9.0
- O		6097	44.9
- AB		416	3.1
Rhesus	v1dab3b_rhesus		
- +ve		11242	82.8
- -ve		2337	17.2

**Table 8.** Results of other standard laboratory tests during pregnancy.

Test	P no.	Q no.	No. tested	No. abnormal
Ketonuria	1076	B6t	8369	864
Urea and electrolytes	1077	B6ll	8369	1029
Alpha-fetoprotein ^a^	1088	B6a	8369	92
Rubella immunity *	1089	B3c	7978	81
Rhesus antibodies	1087	B6aa	8369	29
Other antibodies	1090	B6v	8369	165

*Data also available in extended abstracted dataset; ^a^information on gestation of test and abnormal levels are available.

***Procedures undertaken during pregnancy.*** With the exception of the straightforward ultrasound examinations (see *Ultrasound scans*), the most common procedures undertaken were biophysical profile, amniocentesis and giving anti-D to Rhesus negative women ( Table 9).

**Table 9.** Procedures undertaken during pregnancy, prior to the onset of labour ^a^.

Procedure	P no.	Q no.	No. with information	No. having procedure
Cervical cerclage	1120	B6h	8369	23
Amniocentesis *	1070	B6b	8369	226
Chorionic villus sampling (CVS) *	1071	B6i	8369	62
Biophysical profile	1074	B6e	8369	416
External cephalic version (ECV)	1121	B6n	8369	26
Failed ECV	1122	B6o	8369	38
Anti-D given	1088	B6d	8369	153

*Also available in the full data set; ^a^excluding ultrasound examinations.

***Advice given to the pregnant woman.*** Only specific advice given during pregnancy and written as such within the notes are included in this section ( Table 10). Further information written in the text is likely to expand on, for example, the type of diet recommended.

**Table 10.** Advice given in pregnancy.

Advice	P no.	Q no.	No. with information	No. having advice
Rest in bed for > one week	1123	B9a	8037	50
Rest in bed for < one week	1124	B9b	8038	606
Rest (not bed rest)	1125	B9c	8030	953
Reduce salt in diet	1126	B9d	8073	7
Special diet	1127	B9e	8071	118

***Other details of pregnancy.*** Information on all the various signs and symptoms occurring during pregnancy, together with information on medications taken were collected in three questionnaires completed by the mother and are available to view, with relevant frequencies among the Built Files as the A file (including gestation medication taken in early pregnancy), B file (for data relevant to the first 18 weeks of pregnancy), the C file (for the three months up to 32 weeks gestation), and the D file (for medication regularly taken) (see Ellis *et al.*, 2022). The frequencies of the maternal report of the medication she had taken during pregnancy have also been published ( Headley *et al.*, 2004).

### Labour and delivery

In this section we include aspects relating to the mother but not to the fetus/infant. As a rule of thumb, if the measures for one member of a multiple birth could be different (e.g. presentation at the onset of labour), then the data are described in a further Data Note on the fetus/neonate ( Birmingham *et al.*, 2021d). Although a complete distinction between the two is not feasible, we have tried to minimise the overlap.

In Table 11– Table 14 we describe the various procedures undergone, the methods used to induce and augment labour, the anaesthetics and analgesics used to reduce pain during labour and/or caesarean section, and the other medications administered. 

**Table 11.** The onset and process of labour: hospital admission, membrane rupture, induction, augmentation and caesarean section.

Feature	P no.	Q no.	No. with information	No. with feature
Admitted to hospital *	1140	C2b	8049	7733 admitted from home
Admitted prior to, or in labour	1141	C2a	8163	4077 before labour
How membranes ruptured	1151	C3a	8167	761 at CS; 3795 artificially
Membrane rupture before labour	1154	C3c	8139	2279 before labour
Induction of labour *	1160	C4i	8212	1627 induced
Labour was augmented	1180	C4iiia	7349	4813 augmented
Whether Caesarean section *	1212	C6c	8226	1454 CS: 519 elective; 935 emergency.

*Reasons given as text.

**Table 12.** Drugs and procedures to induce or augment labour.

Drug or procedure	P no.	Q no.	No. with information	No. receiving drug/procedure
*For induction*				
Vaginal prostaglandin gel	1161	C4iia	8369	90
Prostaglandin pessaries	1162	C4iib	8369	1209
Extra-amniotic prostaglandins	1163	C4iic	8369	<5
Oral prostaglandins	1164	C4iid	8369	<5
Artificial rupture of membranes	1165	C4iie	8369	496
Infusion of syntocinon	1166	C4iif	8369	409
Other method *	1167	C4iig	8369	134
*For augmentation*				
Artificial rupture of membranes	1181	C4iiib	7558	3286
Mobilisation of the mother	1182	C4iiic	7558	1925
Syntocinon infusion	1183	C4iiid	7558	1817
Other method *	1184	C4iiie	7558	17

*Described as text. <5 may equal zero.

**Table 13.** Anaesthetics and analgesics administered to mother in labour or at caesarean section.

Painkiller administered	P no.	Q no.	No. with data	No. receiving the substance
*Anaesthetic*				
General anaesthetic	1220	C7ig	8369	651
Lumbar epidural	1221	C7ii	8369	2558
Caudal epidural	1222	C7ib	8369	<5
Epidural not otherwise stated	1223	C7id	8369	36
Perineal infiltration	1224	C7ij	8369	1079
Pudendal block	1225	C7im	8369	254
Spinal anaesthetic	1226	C7in	8369	435
*Analgesics*				
Gas and air	1227	C7if	8369	5623
Diamorphine	1228	C7ic	8369	186
Pethidine	1229	C7ik	8369	2430
Pethilorphan	1230	C7il	8369	<5
Fentanyl	1231	C7ie	8369	412
Birthing pool	1232	C7ia	8369	119
Hot bath/hydrotherapy	1233	C7ih	8369	968
Transcutaneous electrical nerve stimulation (TENS)	1234	C7io	8369	521
Other *	1235	C7ip	8369	1015
No pain relief	1236	C7iq	8369	428

*Described as text. <5 may equal zero.

**Table 14.** Other medication given in labour or at caesarean section.

Medication	P no.	Q no.	No. with information	No. receiving drug
Antibiotics	1240	7iia	8369	128
Diazepam	1241	7iic	8369	5
Nitrazepam	1242	7iif	8369	<5
Temazepam	1243	7iip	8369	84
Phenobarbitone	1244	7iii	8369	8
Phenytoin	1245	7iij	8369	32
Phenergan	1246	7iih	8369	1685
Ranitidine	1247	7iik	8369	1245
Dexamethasone	1248	7iib	8369	5
Dichloralphenazone	1249	7iid	8369	<5
Ephedrine	1250	7iie	8369	412
Ritodrine	1251	7iil	8369	27
Salbutamol	1252	7iim	8369	7
Sodium citrate	1253	7iin	8369	1038
Stemetil	1254	7iio	8369	366
Oxygen	1255	7iig	8369	1713
Other *	1256	7iiq	8369	782
None of the above	1257	7iir	8369	3875

*Described as text. <5 may equal zero.

***Duration of the labour and delivery.*** Many of the details concerning the length of time various birth processes took are shown in Table 15. The variables available include the length of time from membrane rupture to delivery, admission to delivery and the start of labour to delivery. It should be noted that where data are missing, valid information may be available in the STORK dataset (see Mummé *et al.*, 2020).

**Table 15.** Hours of onset and durations of various features of labour and delivery.

Feature	P no.	Q no.	No. with information	Range	Median
Hour of admission	1142	C1ahr	7870	00-23	12 noon
Time from admission to delivery (hr)	1143	C1a,e	7854	-3 to1304	9 hr
Hour of membrane rupture	1150	C1bhr	7360	00-23	10a.m
Rupture of membranes to delivery (hr)	1152	C1b, e	7352	0-1759	4 hr
Hour labour started	1190	C1chr	7278	00-23	12 noon
Length of 1 ^st^ stage of labour (hr)	1191	DV	6701	0-534	6 hr
Length of 2 ^nd^ stage of labour (min)	1192	DV	6783	0-585	30 min
Length of 3 ^rd^ stage of labour (min)	1401	DV	8120	0-275	5 min

***Other noted features of labour.*** There were a variety of different conditions noted in labour, including indications of pre-eclampsia (blood pressure, proteinuria, oedema), haemorrhage, pyrexia, ketonuria and maternal distress. In addition, position in labour as well as whether a water birth or blood transfusion took place ( Table 16). Nevertheless, there were a large number of descriptions written by the data abstractors which have not been coded as yet. They are available to the interested researcher, however. Notably, only 1147 (14%), of the 8369 women with detailed data abstractions had no complications during labour.

**Table 16.** Complications of, and procedures in, labour.

Feature	P no.	Q no.	No. with information	Results
*Hypertension*				
Systolic pressure	1261	C9bsys	6851	Range 60-160; median 130
Diastolic pressure	1262	C9bdia	6860	Range 35-140; median 80
Proteinuria	1264	C10b	3930	Range 0 - +++
Eclampsia	1265	C13f	8369	<5
Oedema present	1266	C12a	5657	376
Sites of oedema	1267	C12b	371	described
*Other conditions*				
Haemorrhage	1268	C8	7488	18 abruption; 8 placenta praevia; 312 non-specific haemorrhage
Pyrexia	1269	C13p	8369	578 pyrexial
Maternal distress	1271	C13e	8369	1076
Ketonuria	1272	C11a	7326	3929 positive
Level	1273	C11b	3910	Range 0 to +++
*Position in labour*				
Left lateral	1300	C13i	8369	2287
Right lateral	1301	C13q	8369	727
*Procedures in labour*				
Water birth	1302	C13r	8369	31
Blood transfusion	1310	C13a	8369	11
Catheterised	1311	C13b	8369	3116
Other complications *	1312	C13s	8369	5525
No complications	1313	C13t	8175	1147

*Described in text <5 may equal zero.

### The mother in the puerperium

***Conditions present in the puerperium.*** The NHS expectation at the time, after the mother had been discharged home, was for the community midwifery team to follow up the mother for the first 14 days after delivery. The midwife’s notes were eventually included in the hospital record folder, and were thence available for scrutiny by the data abstractor, together with those notes made by the clinical team in hospital. From these records, information on the perineal consequences of delivery (episiotomy or tears), postpartum haemorrhage and anaemia were noted, markers of postpartum pre-eclampsia, deep vein thrombosis and pulmonary embolism, and other conditions including infections and procedures such as catheterisation and sterilisation ( Table 17).

**Table 17.** Conditions present in the puerperium.

Condition	P no.	Q no.	No. with information	Results
*Episiotomy, perineal tears, haemorrhages and anaemia*				
Episiotomy	1410	C16a	8197	2197
Perineal tear	1412	C16b2	8195	4243
- 1 ^st^ degree	1411	C16b	8195	550
- 2 ^nd^ degree	1411	C16b	8195	1679
- 3 ^rd^ degree	1411	C16b	8195	112
Post-partum haemorrhage	1420	D2a	8174	1240
- type	1424	D2b	1238	Primary – 1192 Secondary – 33 Both – 13
- amount (ml)	1425	D2c	1199	Range 500-8000 Median 600
Clots passed	1426	D1e	8369	2278
Diagnosed anaemic *	1427	D1a	8369	1295
Lowest Hb level	1428	D1arslt	1289	Range 4.6-9.9 Median 9.1
Blood transfusion	1429	D1b	8369	247
*Cardiovascular markers*				
Systolic blood pressure	1440	D8sys	7983	Range 80-170; median 120
Diastolic blood pressure	1441	D8dia	7995	Range 20-112; median 70
Days after delivery ^a^	1442	dv	7976	Range 0-14; median 3
Eclampsia	1443	D1h	8369	<5
Deep vein thrombosis	1450	D1g	8369	<5
Pulmonary embolism	1451	D1r	8369	<5
*Infections*				
Uterine infection	1470	D1w	8369	165
Genital infection	1471	D1i	8369	75
CS wound infection	1472	D1k	8369	85
Infection of episiotomy or tear	1473	D1l	8369	80
Urinary infection	1474	D1v	8369	162
Pyrexia	1475	D1s	8369	2842
Temperature	1476	D1stemp	2832	Range 37.1-40.5 Median 37.4
Mastitis	1460	D1n	8369	242
*Other conditions and* *procedures*				
Other breast problem ^b^	1461	D1c	8369	5609
Micturition problems	1480	D1o	8369	1254
Catheterisation	1481	D1d	8369	1961
Sterilisation	1615	D1u	8369	63
Haemorrhoids	1482	D1j	8369	1088
Depression	1490	D1f	8369	34
Psychosis	1491	D1q	8369	7
Other disorder ^b^	1500	D1x	8369	7298
No disorder noted	1501	D1y	8121	73

*Also available in the extended abstracted dataset; ^a^ days after delivery the last blood pressure was recorded; ^b^ described as text <5 may equal zero.

***Medications taken postpartum.*** A wide variety of drugs were administered to mothers post-delivery, including painkillers (varying from general anaesthetic and strong opiates to paracetamol), injected drugs to prevent haemorrhage (ergometrine, oxytocin (syntocinon) or a mixture of the two (Syntometrine)), antibiotics and a variety of other medications ( Table 18). Out of the 8369 women, only 31 (0.4%) had no medications at all. It should be noted that mothers also recorded medications they took during this time period (see the E built file). In contrast to the antenatal medication records, these have not yet been coded but are available to the interested researcher.

**Table 18.** Medications taken in the postpartum period.

Medication	P no.	Q no.	No. with Information	No. given the drug
*Painkillers*				
General anaesthetic	1510	D3j	8369	124
Morphine	1513	D3q2	8369	1259
Pethidine	1515	D3t2	8369	109
Omnopon	1517	D3r2	8369	160
Lignocaine	1519	D3n2	8369	1356
Co-dydramol	1521	D3d2	8369	3632
Co-proxamol	1523	D3e2	8369	574
Voltarol	1525	D3z2	8369	1086
Paracetamol	1527	D3s2	8369	4500
*To prevent haemorrhage*				
Ergometrine	1531	D3f2	8368	318
Syntocinon	1533	D3w2	8369	2965
Syntometrine	1535	D3x2	8369	5755
*Other prophylaxis*				
Anti-D	1601	D3b2	8369	808
Mini-pill	1603	D3p2	8369	162
Iron	1593	D3k2	8369	1774
Folic acid	1591	D3h2	8369	217
*Other medications*				
Progesterone	1541	D3u2	8369	136
Fentazin	1551	D3g2	8369	<5
Metoclopramide	1553	D3o2	8369	302
Stemetil	1555	D3v2	8369	757
Temazepam	1557	D3y2	8369	420
Fybogel	1561	D3i2	8369	360
Lactulose	1563	D3m2	8369	1565
Anusol	1565	D3c2	8369	492
Kamillosan	1571	D3l2	8369	713
Witch hazel	1573	D3za2	8369	385
Antibiotics	1581	D3a2	8369	2121
Other drugs *	1611	D3zb	8369	4044
No drugs	1612	D3zc	8369	31

*Described as text. <5 may equal zero.

***Admission and discharge from hospital.*** The women stayed in hospital for a median of three days but a maximum of five weeks post- delivery. Once discharged 222 (2.6%) were readmitted for a variety of reasons ( Table 19). Further details of all women are available as text.

**Table 19.** Hospitalisation(s) post-delivery.

Hospitalisation(s)	P no.	Q no.	No. with information	Results
Duration of stay after delivery (days)	1620	Dv	8054	Range 0–38 Median 3
Place discharged to	1621	D5	8215	Home 7715
Took own discharge	1622	D6	7872	8
Readmitted < six weeks post-delivery *	1623	D7	8187	222

*Reasons keyed as text.

## Discussion and conclusions

This paper demonstrates the wealth of data collected on 8369 pregnancies, selected for a variety of reasons, many of which result in a population weighted with high risk pregnancies; consequently, the pregnancies in this group include all those resulting in fetal/neonatal deaths, multiple births, caesarean sections, preterm deliveries, and cerebral palsy. Care needs to be taken when using these data alone. We are hoping to fill the gaps eventually. When (and if) further data are added, this Data Note will be updated. In the meantime, we have made some suggestions as to ways in which the current data could be analysed.

One of the major reasons why such detailed data were collected concerns the importance of being able to determine the long-term consequences of exposures to the fetus from medications and procedures undertaken during pregnancy as well as the possible consequences to the child of drugs given to the breast-feeding mother. Medications of potential importance include the anaesthetics and analgesics (e.g. opiates including Fentanyl) taken during labour, the drugs used to induce and augment labour (including prostaglandins and synthetic oxytocin), and the analgesic drugs given to the mother postnatally if she was breast feeding. Concerning procedures, long-term effects on the fetus of repeated ultrasound examinations have not, to our knowledge, been considered in the literature. There is some evidence that ultrasound has a warming effect on certain tissues – and in theory this may result in damage to the brain or other organ of the developing fetus. Among the 8369 women, the maximum number of scans recorded was 33. This was considerably above the recommendations at the time.

Little effort has been made to date to examine possible long-term effects although ALSPAC has collected a considerable repertoire of phenotypes on the offspring including neurocognitive, anthropometric, cardiovascular as well as biochemical, genetic and DNA methylation outcomes collected over time – including from the neonatal period through to age 30+.

Additional advantages of the detailed data collection described concern the ability to determine any long-term effects on the mothers themselves, since they have also been followed for 30 years since the study child was delivered. Analyses looking at early outcomes have demonstrated the advantages of the approach (e.g. Martin *et al.*, 2022; Patel *et al.*, 2007), but long-term documentation has yet to be undertaken. 

It may be claimed that the pregnancies of 30 years ago will have no relevance today. However, there are two reasons for looking at the long-term effects of the features of the treatment of the mother as shown here: (i) even if a substance/procedure is no longer used, any long term effects (positive or negative) will add to basic biological knowledge, and (ii) if still being used, the study of these data will add to overall knowledge as to their safety (or the risks attached to their use) throughout the lives of the mother and her offspring.

The data described in this Data Note are confined to what was documented in the medical record. It is important to point out, however, that other relevant data are available including the computerised STORK data ( Mummé *et al.*, 2020), and the prospective data collected from the women during pregnancy and after delivery, using detailed self-report questionnaires administered at 18 and 32 weeks gestation as well as at eight weeks postpartum ( Ellis *et al.*, 2022). These provide information on a variety of signs and symptoms occurring during and after the pregnancy, together with the non-obstetric medications taken.

### Strengths and limitations

 There are four major strengths of these data. Firstly, each item was abstracted from the paper medical record with a strict protocol and meticulous checking; secondly, the data collected were documented at the time that the maternal examinations were undertaken, the treatments prescribed and the observations made, so that there was no element of retrospective recall; thirdly, these data can be augmented by information from the maternal questionnaires completed prenatally and postnatally on her signs and symptoms, procedures undertaken and medications taken, to provide a different aspect of the pregnancy; and fourthly, and importantly, the data provide an important baseline from which to assess the long-term benefits and possible hazards of the various facets of maternity care.

 There is one major limitation of the data – which is that, with the exception of the important longitudinal information on blood pressure, weight and diabetes, the details on many aspects of maternal exposures and conditions are missing for over 5000 pregnancies. Admittedly, by the selection criteria used on the 8369, the majority of the more complex pregnancies have already been abstracted, but for valid epidemiological analysis the population of all ‘normal’ pregnancies are also needed. It is hoped that efforts can be made in the future to fill this important gap. 

## Data Availability

ALSPAC data access is through a system of managed open access. The steps below highlight how to apply for access to the data included in this data note and all other ALSPAC data: 1. Please read the ALSPAC access policy which describes the process of accessing the data and samples in detail, and outlines the costs associated with doing so. 2. You may also find it useful to browse our fully searchable research proposal database which lists all research projects that have been approved since April 2011. 3. Please submit your research proposal for consideration by the ALSPAC Executive Committee. You will receive a response within 10 working days to advise you whether your proposal has been approved. Figshare: ALSPAC Mother during pregnancy and the puerperium_data abstraction form. https://doi.org/10.6084/m9.figshare.13614701 ( Birmingham *et al.*, 2021a). Figshare: ALSPAC Mother during pregnancy and the puerperium_data abstraction instructions. https://doi.org/10.6084/m9.figshare.13621598 ( Birmingham *et al.*, 2021b). Figshare: ALSPAC Mother during pregnancy and the puerperium_ abstraction checking instructions. https://doi.org/10.6084/m9.figshare.13621703 ( Birmingham *et al.*, 2021c). Data are available under the terms of the Creative Commons Attribution 4.0 International license (CC-BY 4.0).

## Footnotes

**1** The website will take you to the large Data Dictionary. Download this (it will take a couple of minutes) and select ‘Built files’; then select ‘Other’, then ‘Obstetric’ and then D4200_OA.

**2** The website will take you to the large Data Dictionary. Download this (it will take a couple of minutes) and select ‘Built files’; then select ‘Other’, then ‘Obstetric’ and then D4201_OB.
