# Supplementary material for: The mother during pregnancy and the puerperium: Detailed data abstracted from the clinical obstetric records of ALSPAC pregnancies
Source: Wellcome Open Res. 2024 Mar 26;6:41. Originally published 2021 Feb 23. [Version 2] doi: 10.12688/wellcomeopenres.16603.2 (PMC11208852; doi:10.12688/wellcomeopenres.16603.2)
Supplement: Peer review history

# Reviewer report: Reviewer response for version 2

**DOI:** 10.21956/wellcomeopenres.23365.r77719

**Published:** 2024-08-05

**Authors:** Wojciech Hanke

**Affiliations:** 1 Nofer Institute of Occupational Medicine, Łódź, Poland

I accept all changes made in the article. I do not have any further comments.

Are sufficient details of methods and materials provided to allow replication by others?

Yes

Is the rationale for creating the dataset(s) clearly described?

Yes

Are the datasets clearly presented in a useable and accessible format?

Yes

Are the protocols appropriate and is the work technically sound?

Yes

Reviewer Expertise:

Environmental epidemiology

I confirm that I have read this submission and believe that I have an appropriate level of expertise to confirm that it is of an acceptable scientific standard.

# Reviewer report: Reviewer response for version 2

**DOI:** 10.21956/wellcomeopenres.23365.r84418

**Published:** 2024-07-05

**Authors:** K S Joseph

**Affiliations:** 1 University of British Columbia, Columbia, Canada

The article provides details regarding information abstracted from the obstetric medical records of mothers included in the Avon Longitudinal Study of Parents and Children (ALSPAC). The records cover pregnancy, labour, delivery and the first two weeks of the puerperium. This description of the cohort is intended to facilitate exploitation of the collected data for research into the perinatal antecedents of developmental and long-term outcomes for both mothers and babies. Recent publications based on the 50-year follow up the Collaborative Perinatal Project attest to the utility of such endeavors (see Hinkle *et al*. Lancet 2023).

 As mentioned by the authors, the initial ALSPAC sample consisted of 14,541 pregnancies, 14,062 live births and 13,988 children who were alive at one year of age. This is a reasonably large population that included 80% of eligible pregnant women. The documented experience of these women and the obstetric care they received in 1990-92 (including the medications, ultrasound exposure, pregnancy complications, etc) could provide scientific insights to questions that may or may not be obvious today. 

 Data collected on some maternal factors (such as blood groups, weight, blood pressure, glycosuria, haemoglobin, proteinuria and related derived variables) are available on a total of 13,706 women (13,899 offspring). However, complete information from medical records is currently available for only a sample of 8,369 pregnancies. The availability of information on a selected (non-random) subset of the cohort will present a challenge to some researchers. This limitation is acknowledged clearly by the authors and it is hoped that availability of subsequent funding will permit addition of information on the remaining 5,336 pregnancies. Meanwhile, a clear understanding of the process by which records were selected for inclusion in the subsample (of 8,369 pregnancies) will allow researchers to use these data for answering specific questions in a valid manner.

 Open access to the data from cohorts such as  ALSPAC will enhance and facilitate scientific studies on clinical and epidemiologic issues. The authors are to be applauded for their efforts to make these data available for wider exploitation, and it is hoped that their current and future attempts to abstract information on the complete cohort are successful.

Are sufficient details of methods and materials provided to allow replication by others?

Yes

Is the rationale for creating the dataset(s) clearly described?

Yes

Are the datasets clearly presented in a useable and accessible format?

Yes

Are the protocols appropriate and is the work technically sound?

Yes

Reviewer Expertise:

Perinatal epidemiology; Pregnancy complications; Preterm birth and growth restriction; Maternal morbidity and mortality; Perinatal mortality and severe neonatal morbidity.

I confirm that I have read this submission and believe that I have an appropriate level of expertise to confirm that it is of an acceptable scientific standard.

# Reviewer report: Reviewer response for version 2

**DOI:** 10.21956/wellcomeopenres.23365.r84422

**Published:** 2024-06-28

**Authors:** James Roberts

**Affiliations:** 1 Magee-Womens Research Institute, University of Pittsburgh, Pittsburgh, PA, USA

In general, the ALSPAC database is a remarkable resource despite some limitations. It was drawn from 14,000 pregnancies occurring between the 1st of April of 1991 and the 31st of December 1992. The strengths are the enormous amount of data that is available, and the excellent collection and checking strategies. It also seems that the information that has not been collected thus far has at least the possibility of being extracted from stored information in the future. Based on their ability to obtain funding they were able to complete the planned data assessment of about 8,000 of the 14,000 pregnancies that are available. They also, were able to augment the 8,000 plus patients with another 5,000 with some but not all the data that they had originally hoped to collect. This has been further augmented by other studies which examined certain variables in that were extracted for particular studies. In addition, the collection of the data, especially that of the 1st 8,000 patients is more heavily weighted towards pregnancies that are not normal. This is less so in the additional 4,000 pregnancies. They point out strategies to circumvent these limitations.

 The weaknesses of the study are largely the failure to have a complete set of data on all patients. There is the hope in the future to extend the amount of data to include more normal patients in the future. Although it has never stated explicitly it seems that all the data which has not been collected is in theory potentially collectible in future studies. It would be helpful if this point had been made more clearly. The other evident weakness of the data is the fact that it was collected many years ago. This is, nonetheless, also a strength. The weakness is that obstetrical care patterns have changed, and general population characteristics (for example the frequency of obesity) have changed over time. However, although again not stated explicitly they apparently have the capacity to follow these mothers and infants even at this long time after pregnancy (it would be useful to make this clearer). That length of follow up to examine the impact of the huge amounts of data describing pregnancy and outcomes is available in almost no other setting.

 In summary the database is a remarkable resource. It has limitations, but it does appear that many of these limitations can still be potentially overcome if sufficient funding was available. However, even in the form it exists it has remarkable amount of information well collected over a remarkable time span to answer many very important questions.

Are sufficient details of methods and materials provided to allow replication by others?

Yes

Is the rationale for creating the dataset(s) clearly described?

Yes

Are the datasets clearly presented in a useable and accessible format?

Yes

Are the protocols appropriate and is the work technically sound?

Yes

Reviewer Expertise:

Pregnancy, adverse outcomes in pregnancy, preeclampsia

I confirm that I have read this submission and believe that I have an appropriate level of expertise to confirm that it is of an acceptable scientific standard.

# Reviewer report: Reviewer response for version 2

**DOI:** 10.21956/wellcomeopenres.23365.r81288

**Published:** 2024-06-26

**Authors:** Jan van der Meulen

**Affiliations:** 1 London School of Hygiene & Tropical Medicine, London, UK

ALSPAC provides a fascinating example of an attempt to create an environment for long-term follow-up of mothers and their babies born in a defined geographical area.

 I've got a number of comments:

 1) It is baffling to read that abstraction of the records of babies born in the early 1990s is still ongoing and even more so that data abstraction in itself can take up to two hours per women. An unanswered question is whether the authors expect that another 10,000 hours will need to be spent on data abstraction for the more than 5,000 women who together with their babies are still not included in the ALSPAC cohort and when this will be completed. Related to this, I don't feel that the authors present enough evidence that this ongoing effort is still relevant.

 2) I read little about the completeness and accuracy of the abstracted data. I assume that the approach was taken to record "abnormalities"' that were mentioned in the medical notes and that not being able to find a mention of these abnormalities indicates that these abnormalities were not present. This is a potentially problematic feature. In this context, I also have to indicate that I disagree with the statement of the authors that "there was no element of retrospective recall". All data collected from medical notes is "retrospective".

 3) It read very little about actual data checking and validation, apart from what can be done around the data input processes itself. We have to accept that data abstraction from medical notes will have limitations (i.e., typically data completeness and quality inherently depend on the quality of the notes themselves) that cannot be overcome during the abstraction.

 4) Given the ongoing developments of obstetric care and support for women before, during and after pregnancy, there is also a question about the relevance of some reported clinical findings. The ultrasound results are a key example. I'm no expert but it is likely that ultrasound examination will have 'under-detected' abnormalities compared to ultrasound examination today.

 4) A question that I think needs a bit more attention is the possible impact of selective inclusion of mothers and their babies as well as selective missingness of data. What work has been done to assess their impact and also what are the approaches to overcome potential selection bias issues?

 5) I would have liked to see a more detailed description of the data linkages that have been undertaken. Now, they are mentioned only once and that is a core admission, especially given that so much space has been assigned to details of abstracted data items that may be less relevant than the potential that data linkage can offer.

 6) A critical reflection on what ALSPAC teaches about the future of longitudinal studies in the first place would have been welcome, especially given the move towards secondary use of routinely collected electronic data, data linkage and so on.

Are sufficient details of methods and materials provided to allow replication by others?

No

Is the rationale for creating the dataset(s) clearly described?

Yes

Are the datasets clearly presented in a useable and accessible format?

No

Are the protocols appropriate and is the work technically sound?

Partly

Reviewer Expertise:

Clinical epidemiology, data science, medical statistics, maternity, women's health cancer, healthcare performance assessment

I confirm that I have read this submission and believe that I have an appropriate level of expertise to confirm that it is of an acceptable scientific standard, however I have significant reservations, as outlined above.

# Reviewer report: Reviewer response for version 1

**DOI:** 10.21956/wellcomeopenres.18300.r67060

**Published:** 2024-03-25

**Authors:** Wojciech Hanke

**Affiliations:** 1 Nofer Institute of Occupational Medicine, Łódź, Poland

The paper describes the process of obtaining information about the clinical aspects of labor to appropriately describe obstetrics health outcomes.

 The descriptions are generally clear and allow for a detailed methodology presentation.

 The only information, which might need some clarification is the one related to the definition of preterm delivery. As I understand it was based on a criterion of of less than 37 weeks of gestation. Has there been any effort to distinguish spontaneous preterm delivery from the one resulting from medical indications?

Are sufficient details of methods and materials provided to allow replication by others?

Yes

Is the rationale for creating the dataset(s) clearly described?

Yes

Are the datasets clearly presented in a useable and accessible format?

Yes

Are the protocols appropriate and is the work technically sound?

Yes

Reviewer Expertise:

Environmental epidemiology

I confirm that I have read this submission and believe that I have an appropriate level of expertise to confirm that it is of an acceptable scientific standard.

# Reviewer report: Reviewer response for version 1

**DOI:** 10.21956/wellcomeopenres.18300.r43325

**Published:** 2022-05-03

**Authors:** Tamar Wainstock

**Affiliations:** 1 Department of Public Health, Faculty of Health Sciences, Ben-Gurion University of the Negev, Beer-Sheva, Israel

This manuscript describes a prospective cohort study, including prenatal data regarding a large sample of mother-offspring dyads. Besides the relatively detailed information regarding the available data and the means of data collection, the authors propose to make this data available for researchers to analyze. Although there is very detailed information regarding the perinatal period, the external validity of the findings based on this cohort may be limited since this data was collected decades ago. Regarding the long term effects of certain early life exposures, it is unclear what long-term data is available for research, regarding both the mother and the offspring.

Are sufficient details of methods and materials provided to allow replication by others?

Yes

Is the rationale for creating the dataset(s) clearly described?

Yes

Are the datasets clearly presented in a useable and accessible format?

Partly

Are the protocols appropriate and is the work technically sound?

Yes

Reviewer Expertise:

Perinatal and environmental epidemiology

I confirm that I have read this submission and believe that I have an appropriate level of expertise to confirm that it is of an acceptable scientific standard, however I have significant reservations, as outlined above.

# Response

**Published:** 2022-05-12

**Authors:** Yasmin Iles-Caven

**Affiliations:** University of Bristol, UK

Dr Wainstock correctly points out that the information described was collected long ago. It is true that obstetric practice may have changed over time but, we feel that  in many respects the data are likely to provide useful information of relevance to the present day. Examples still of likely relevance include pre-eclampsia and placenta praevia. Of course there is the conundrum that if there are important long-term effects, they are unlikely to be evident for many years, by which time the particular exposure in question may not be in use. Nevertheless, it is important to document any long-term associations as they may be important to scientific understanding in the future.

 The long-term outcomes in the mothers and children who have taken part in the ALSPAC study are continuously updated. Details can be retrieved from the ALSPAC website: Explore data and samples | Avon Longitudinal Study of Parents and Children | University of Bristol. Information available includes medical and psychological outcomes, genetic and epigenetic data as well as the results of various psychometric and anthropometric measurements. The developing database includes details of the offspring of the original study children, thus enabling research into transgenerational associations.

# Response

**Published:** 2024-02-16

**Authors:** Yasmin Iles-Caven

**Affiliations:** University of Bristol, UK

Further to our previous comments, in regard to the points made concerning the fact that the data are out of date, we have updated the paper and added further explanation to the end of the Discussion concerning the probable relevance of the data to the analysis of factors contributing to the health of both the mothers and their offspring long-term.

# Response

**Published:** 2024-04-22

**Authors:** Wojciech Hanke

**Affiliations:** Department of Environmental Epidemiiology, Institute of Occupational Medicine, Lodz, POland, Poland

The present data is unique information addressing the health and the mother's situation during and after delivery. However, to assess the relevance of the presented information, it is necessary to have a clear goal for the manuscript, which I can not find.
